# Causal relationship between gut microbiota and pyogenic arthritis: a two-sample Mendelian randomization study

**DOI:** 10.1099/jmm.0.002004

**Published:** 2025-04-15

**Authors:** Ji-Ang Li, Chen-Han Zhou, Han-Dan Xiao, Hong-Bin Guo, Jie-Yu Liang, Yi Zhang

**Affiliations:** 1 Department of Orthopaedics, Xiangya Hospital, Central South University, Changsha 410017, Hunan Province, PR China; 2 National Clinical Research Center for Geriatric Disorders, Xiangya Hospital, Central South University, Changsha 410017, Hunan Province, PR China; 3 Department of Radiology, Xiangya Hospital, Central South University, Changsha 410017, Hunan Province, PR China

**Keywords:** causal inference, gut microbiota, Mendelian randomization (MR) study, pyogenic arthritis (PA)

## Abstract

**Introduction.** Accumulating evidence indicates a significant association between gut microbiota and the risk of developing pyogenic arthritis (PA). However, their causal relationship has yet to be elucidated.

**Hypothesis.** The gut microbiota is causally associated with the risk of PA.

**Aim.** The Mendelian randomization (MR) methodology was employed to assess the potential causal effects of gut microbiota on the susceptibility to PA.

**Methodology.** A two-sample MR study was performed using the summary statistics of gut microbiota from the largest available genome-wide association study meta-analysis (*n*=13,266) conducted by the MiBioGen consortium. The summary statistics of PA were obtained from the R11 release data provided by the FinnGen consortium (2,441 cases and 2,87,796 controls). Inverse-variance weighted (IVW) model, weighted median estimator model, weighted model-based method and MR-Egger regression (MER) model were used to examine the causal association between gut microbiota and PA. To assess the heterogeneity and pleiotropic effects of the identified instrumental variables (IVs), we utilized several analytical methods, including the leave-one-out sensitivity analysis, the MR Pleiotropy Residual Sum and Outlier test and Cochran’s Q test.

**Results.** Utilizing the IVW method, we identified six bacterial traits that were negatively correlated with PA: *Eubacterium eligens* group [OR: 0.6057; 95 % confidence interval (CI): 0.4525 to 0.8107; *P*=0.0007], *Barnesiella* (OR: 0.7456; 95 % CI: 0.5760 to 0.9651; *P*=0.0258), *Coprococcus2* (OR: 0.7257; 95 % CI: 0.5352 to 0.9840; *P*=0.0391), *Ruminococcaceae* UCG005 (OR: 0.7562; 95 % CI: 0.5920 to 0.9660; *P*=0.0252), *E. oxidoreducens* group (OR: 0.7311; 95 % CI: 0.5547 to 0.9637; *P*=0.0262) and *Lachnospiraceae FCS020* group (OR: 0.7825; 95 % CI: 0.6135 to 0.9981; *P*=0.0482), respectively. On the contrary, four bacterial traits were positively correlated with PA: *Adlercreutzia* (OR 1.3210, 95 % CI 1.0181–1.7141, *P*=0.0362), *Holdemania* (OR 1.2239, 95 % CI 1.0013–1.4960, *P*=0.0485), *Anaerostipes* (OR 1.3614, 95 % CI 1.0189–1.8191, *P*=0.0369) and *Butyricimonas* (OR 1.2627, 95 % CI 1.0016–1.5921, *P*=0.0484), respectively. No significant heterogeneity among IVs or evidence of horizontal pleiotropy was detected.

**Conclusion.** Our research demonstrates a potential causal link between various gut microbiota and the risk of PA. Further research is imperative to elucidate the mechanisms by which gut microbiota influence the pathogenesis of PA.

## Data Availability

The original contributions presented in the study are included in the article/Supplementary Material. Further inquiries can be directed to the corresponding author.

## Introduction

Pyogenic arthritis (PA), alternatively referred to as septic arthritis, constitutes a critical infectious disorder impacting joints (swift and permanent joint damage if addressed not timely) [1,2]. The worldwide incidence of PA exhibits variability, with annual estimates ranging from 4 to 10 occurrences per 1,00,000 individuals [3]. However, the rate escalates markedly due to immunosuppression, chronic joint disorders and diabetes mellitus [4]. Due to the swift progression and severe outcome, PA is regarded as a medical crisis, posing a substantial challenge to healthcare systems [3]. The primary aetiology of PA is attributed to circulating bacterial pathogens, with *Staphylococcus aureus* being the predominant causative agent [5]. However, emerging evidence suggests that the systemic factors, such as gut microbiota, may also contribute to the susceptibility to PA [6,7].

The human gut microbiota is a diverse ecosystem of microorganisms that play an essential role in immune regulation and systemic inflammation [6,8, 9]. Dysbiosis, an imbalance in gut microbiota composition, has been implicated in a range of inflammatory and infectious diseases (rheumatoid arthritis and inflammatory bowel disease) [7,10, 11]. Interestingly, dysbiosis was also reported to influence immune responses and increase susceptibility to arthritis [8]. An imbalance in the gut microbiota can trigger an immune response characterized by the production of antibodies upon antigen stimulation, which may lead to the erroneous targeting of body tissues by autoantibodies, including joints and skin [8]. In addition, pathogens can exploit gut microbiota to enhance their transmission efficiency in certain cases [8]. However, the current evidence is limited in observational study design and small sample size (susceptible to potential confounding factors and reverse causation), which preclude a clear causal relationship between gut microbiota and PA.

Nevertheless, Mendelian randomization (MR) offers a promising approach to assess the causal relationship between gut microbiota and PA. MR utilizes genetic variants as instrumental variables (IV) to test the effect of an exposure on an outcome [12]. Genetic variants are randomly assigned during meiosis and are independent of lifestyle or environmental factors, which consequently reduces the risk of confounding and reverse causality [13]. For instance, single nucleotide polymorphisms (SNPs) associated with gut microbiota traits identified through genome-wide association studies (GWAS) can be used as proxies to assess whether variations in the gut microbiota are causally linked to PA risk. In light of the limitations of current evidence, the present study aims to utilize a two-sample MR approach to explore the causal relationship between gut microbiota composition and the risk of PA. By employing MR analysis, this study minimizes confounding factors and establishes a robust causal relationship between gut microbiota and PA. This methodological rigour supports the reliability of findings, paving the way for clinical applications.

## Method

### Data sources

Genetic variants associated with the gut microbiota were extracted from the most comprehensive genome-wide meta-analysis available to date, executed by the MiBioGen consortium [14]. The research incorporated data from 18,340 participants across 24 cohorts, with the majority being of European descent (*n*=13,266) and ages ranging from 4 to 89 years old, including individuals from the United States, Canada, Israel, South Korea, Germany, Denmark, the Netherlands, Belgium, Sweden, Finland and the United Kingdom. The study focused on profiling the microbial composition by targeting the V4, V3–V4 and V1–V2 variable regions of the 16S rRNA gene. This profiling strategy involved direct taxonomic binning for taxonomic classification. To pinpoint host genetic variants linked to the abundance of bacterial taxa in the gut microbiota, microbiota quantitative trait loci mapping analysis was employed [14]. In this research, the genus represented the most specific taxonomic category. A total of 131 genera were identified, each exhibiting an average abundance exceeding 1%, among which 12 genera remained unidentified [14]. Consequently, the analysis incorporated 119 taxa at the genus level. The PA-related GWAS summary statistics were derived from the R11 release data provided by the FinnGen consortium. In the present study, the phenotype designated as ‘pyogenic arthritis’ was utilized. This GWAS encompassed a cohort of 2,90,237 Finnish adults, comprising 2,441 cases and 2,87,796 controls.

### Selection of instrumental variables

**Fig. 1.**
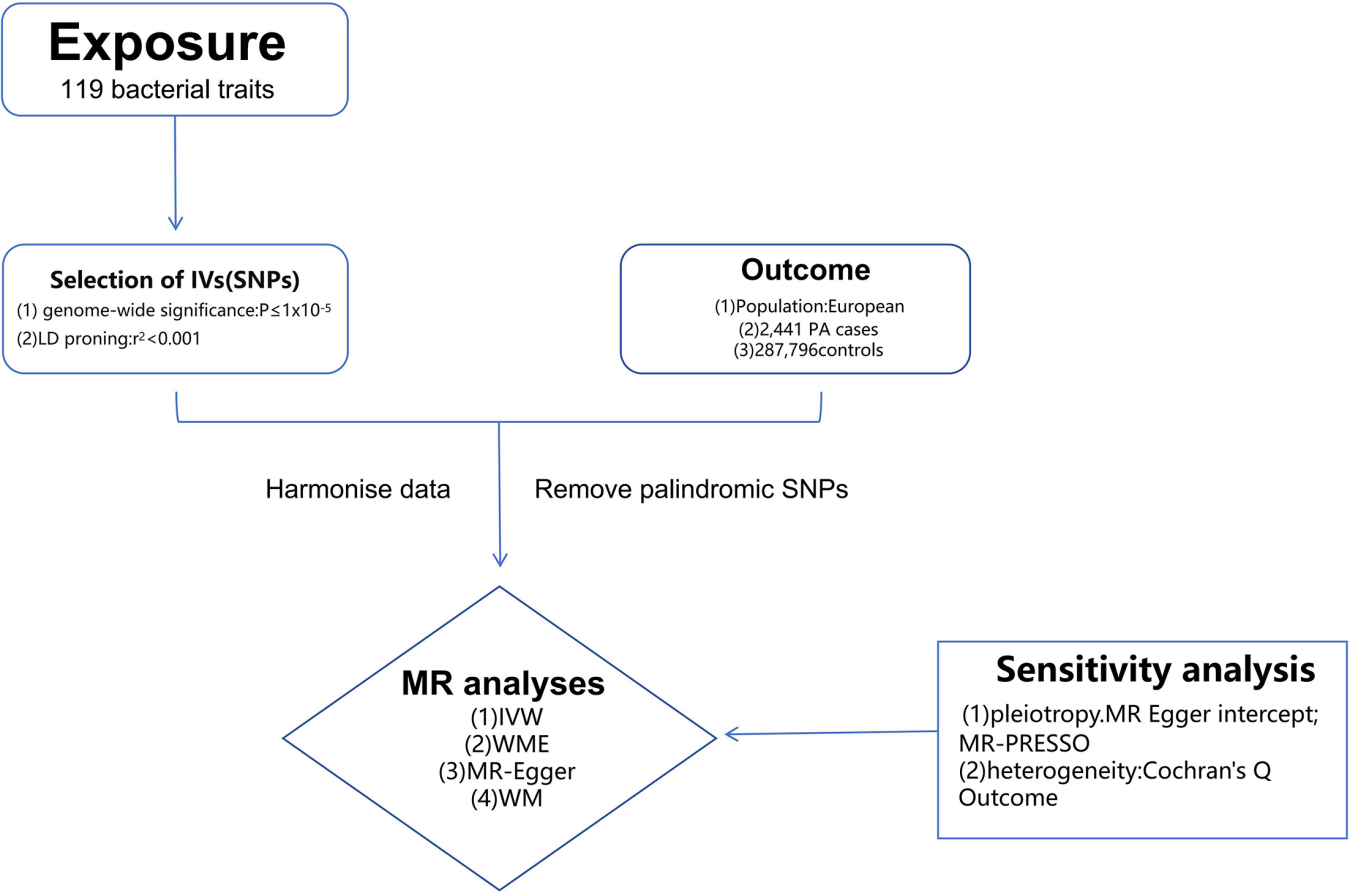
A flow diagram depicting the procedures of the MR analysis for examining the relationship between gut microbiota and PA.

To ensure the robustness of the MR analysis, it is imperative to satisfy three fundamental assumptions: (a) the IV must exhibit a strong association with the exposure factor; (b) the IV should remain unaffected by potential confounding variables; and (c) the outcome should be influenced by the IV exclusively through the exposure factor [14]. Initially, we identified IVs using a significance threshold of *P*<1.0×10^−5^. Then, palindromic A/T or G/C alleles were excluded. Afterwards, to ensure independence, SNPs within each bacterial taxon were clumped. The linkage disequilibrium (LD) threshold for clumping was set to r2<0.001, with a clumping window size of 10,000 kb (Fig. 1). LD was estimated based on the European-based 1000 Genome Projects reference panel. A total of 1,531 independent SNPs were found to be associated with 119 bacterial traits (Tables S1 and S2, available in the online version of this article). Finally, the strength of the instruments was quantified by calculating F-statistics using the formula F = R^2^ (N-k-1)/k (1-R^2^), where R^2^ represents the proportion of variance in the exposure explained by genetic variants, N denotes the sample size and k is the number of instruments. An F-statistic greater than 10 indicated the absence of significant weak instrumental bias [15].

### Statistical analysis

To assess potential causal relationships between gut microbiota and PA, we utilized a comprehensive array of analytical techniques, such as the fixed/random-effects inverse-variance weighted (IVW) method, the weighted median estimator (WME) model, MR-Egger regression (MER) and the weighted model-based (WM) approach. The IVW method, employing a meta-analytic approach to synthesize Wald estimates for each IV in the absence of directional pleiotropy, produced a robust and precise causal inference [12]. The precision of the results was maximized when horizontal pleiotropy was absent. To assess heterogeneity, we employed Cochran’s Q test. Upon detecting statistically significant heterogeneity (*P*<0.05), we adopted the multiplicative random effects model; otherwise, we utilized the fixed effects model. The WME demonstrated robust performance when more than 50% of the weights were associated with invalid IVs. In the presence of horizontal pleiotropy, the type I error rate was reduced, facilitating a more precise assessment of causal associations [16]. The WM method achieved a robust overall causal estimate provided the majority of similar individual estimates stemmed from valid IVs [17]. Crucially, the MER method yielded a consistently robust estimate regardless of the validity of IVs. Significantly, a non-zero intercept in the MER highlighted the presence of an average horizontal pleiotropic effect among the genetic variants [18,19]. However, it is important to consider that the WME, WM and MER methods exhibit reduced power due to wide confidence intervals (CIs) [20]. Therefore, these methods should be regarded as complementary to the IVW method.

Directional pleiotropy was assessed and corrected utilizing the intercept obtained from the MER analysis, alongside the MR Pleiotropy RESidual Sum and Outlier (MR-PRESSO) test [19]. Furthermore, a subsequent distortion test was conducted to evaluate the effects of outlying IVs identified by the MR-PRESSO tests. Any outliers found in the distortion test with a *P* value below 0.05 were excluded, and the causal estimates were re-evaluated [21]. Besides, a ‘leave-one-out’ analysis was implemented by sequentially excluding each instrumental SNP to identify possible heterogeneous SNPs. To manage multiple testing and minimize the probability of false-positive results, the false discovery rate is employed, maintaining a threshold of q-value less than 0.1 [22]. A suggestive association between genera of gut microbiota and PA is considered when *P*<0.05 but q≥0.1. The MR analyses were carried out using ‘Two-Sample MR’ and ‘MRPRESSO’ packages in R software (version 4.4.1).

## Result

### The main results of the 119 bacterial traits with the risk of PA

**Fig. 2.**
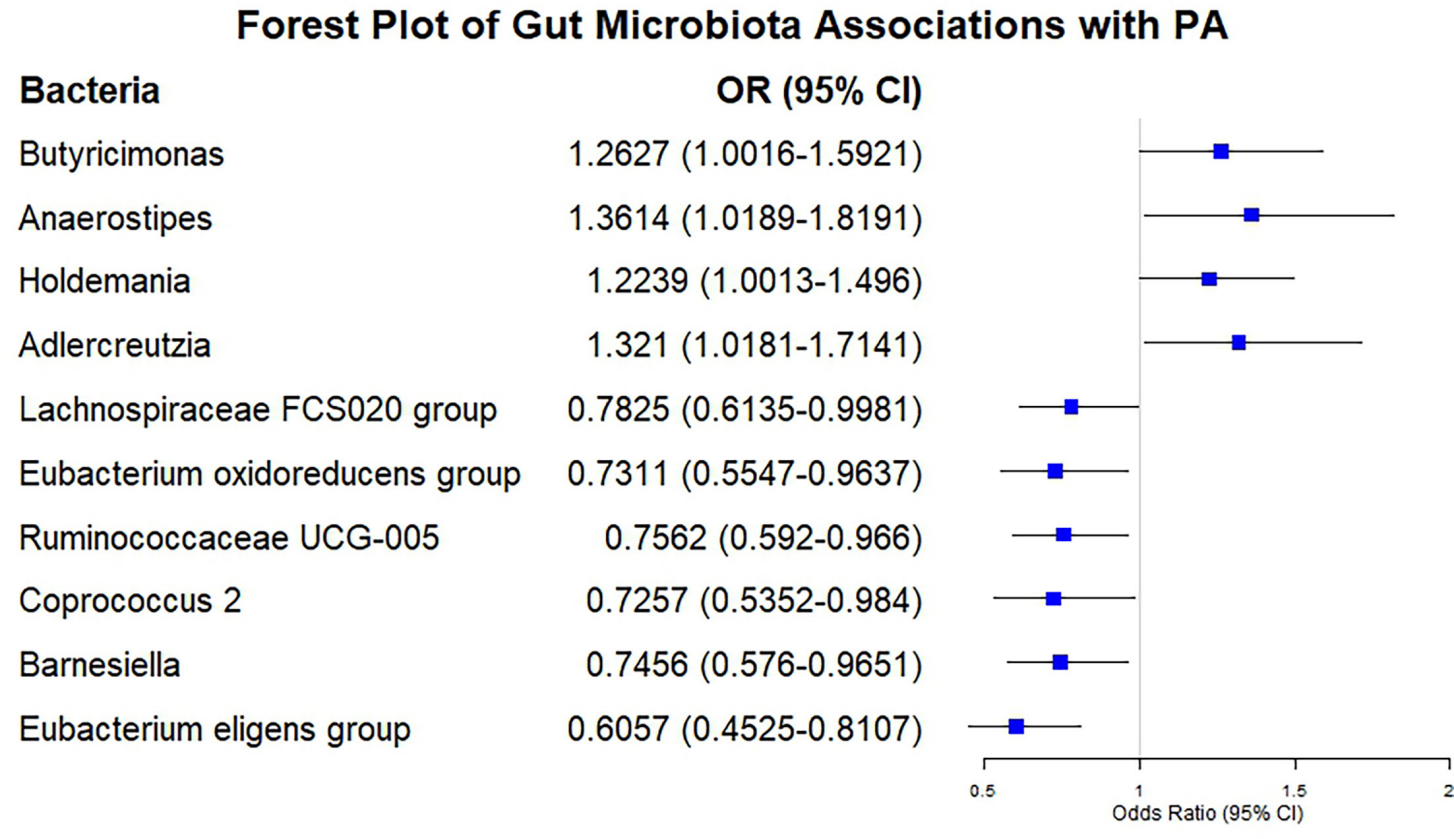
Forest Plot of Gut Microbiota Associations with PA risk.

The F-statistics for all 119 bacterial traits exceeded the threshold of 10, suggesting a diminished probability of weak instrument bias. The detailed results on the associations between all 119 bacterial traits and risk of PA were presented in Table S3. The MR analysis identified significant associations between specific gut microbiota genera and the risk of PA. Six bacterial genera were negatively associated with PA risk, indicating potential protective effects, while four genera showed a positive association, suggesting increased susceptibility (Fig. 2).

### Six bacterial traits are negatively correlated with PA

The IVW method found a positive association between four gut bacteria and PA risk: *Eubacterium eligens* group (OR: 0.6057; 95 % CI: 0.4525 to 0.8107, *P*=0.0007, q=0.0891), *Barnesiella* (OR: 0.7456; 95 % CI: 0.5760 to 0.9651, *P*=0.0258, q=1.0000), *Coprococcus2* (OR: 0.7257; 95 % CI: 0.5352 to 0.9840, *P*=0.0391, q=0.6644), *Ruminococcaceae* UCG005 (OR: 0.7562; 95 % CI: 0.5920 to 0.9660, *P*=0.0252, q=1.0000), *E. oxidoreducens* group (OR: 0.7311; 95 % CI: 0.5547 to 0.9637, *P*=0.0262, q=0.7817) and *Lachnospiraceae FCS020* group (OR: 0.7825; 95 % CI: 0.6135 to 0.9981, *P*=0.0482, q=0.7174), respectively. The results are presented in Table 1 in detail.

**Table 1.** The MR results of six gut microbiota that were identified to be negatively correlated with PA

Exposure	Method	nSNP	b	se	pval	lo_ci	up_ci	OR	or_lci95	or_uci95	Egger_intercept	SE	Pval	Q	Q_df	Q_pval
***Barnesiella***	MR Egger	16	−0.3601	0.5878	0.5499	−1.5122	0.7919	0.6976	0.2204	2.2077	0.0053	0.0457	0.9089			
	Weighted median	16	−0.4582	0.1817	0.0117	−0.8143	−0.1022	0.6324	0.4430	0.9029						
	**Inverse variance weighted**	**16**	**−0.2935**	**0.1317**	**0.0258**	**−0.5516**	**−0.0354**	**0.7456**	**0.5760**	**0.9652**				15.7176	15.0000	0.4011
	Weighted mode	16	−0.6039	0.3519	0.1067	−1.2936	0.0859	0.5467	0.2743	1.0896						
***Coprococcus* 2**	MR Egger	9	−1.2207	0.8200	0.1802	−2.8279	0.3866	0.2950	0.0591	1.4719	0.0691	0.0618	0.3005			
	Weighted median	9	−0.3405	0.2072	0.1003	−0.7465	0.0656	0.7114	0.4740	1.0677						
	**Inverse variance weighted**	**9**	**−0.3205**	**0.1553**	**0.0391**	**−0.6250**	**−0.0161**	**0.7258**	**0.5353**	**0.9841**				3.8753	8.0000	0.8682
	Weighted mode	9	−0.3881	0.3408	0.2878	−1.0562	0.2799	0.6783	0.3478	1.3230						
***Eubacterium eligens group***	MR Egger	10	−1.0950	0.4077	0.0277	−1.8942	−0.2958	0.3345	0.1504	0.7439	0.0610	0.0390	0.1565			
	Weighted median	10	−0.5345	0.2076	0.0100	−0.9414	−0.1276	0.5860	0.3901	0.8802						
	**Inverse variance weighted**	**10**	**−0.5014**	**0.1487**	**0.0007**	**−0.7929**	**−0.2099**	**0.6057**	**0.4525**	**0.8107**				8.3795	9.0000	0.4964
	Weighted mode	10	−0.6458	0.3595	0.1060	−1.3504	0.0588	0.5242	0.2591	1.0605						
***Eubacterium oxidoreducen*s group**	MR Egger	6	−0.2870	0.6239	0.6694	−1.5099	0.9359	0.7505	0.2209	2.5495	−0.0028	0.0651	0.9676			
	Weighted median	6	−0.2791	0.1798	0.1206	−0.6314	0.0733	0.7565	0.5318	1.0760						
	**Inverse variance weighted**	**6**	**−0.3131**	**0.1409**	**0.0263**	**−0.5893**	**−0.0369**	**0.7312**	**0.5547**	**0.9637**				5.8393	5.0000	0.3222
	Weighted mode	6	−0.2136	0.2873	0.4907	−0.7767	0.3496	0.8077	0.4599	1.4185						
***Lachnospiraceae* FCS020 group**	MR Egger	15	−0.4460	0.3292	0.1986	−1.0913	0.1992	0.6402	0.3358	1.2205	0.0171	0.0259	0.5216			
	Weighted median	15	−0.2081	0.1659	0.2098	−0.5332	0.1171	0.8122	0.5867	1.1242						
	**Inverse variance weighted**	**15**	**−0.2452**	**0.1241**	**0.0482**	**−0.4885**	**−0.0019**	**0.7825**	**0.6135**	**0.9981**				9.5944	14.0000	0.7912
	Weighted mode	15	−0.1969	0.2934	0.5131	−0.7720	0.3782	0.8213	0.4621	1.4596						
***Ruminococcaceae* UCG005**	MR Egger	17	−0.0400	0.3511	0.9107	−0.7282	0.6482	0.9607	0.4828	1.9120	−0.0215	0.0294	0.4760			
	Weighted median	17	−0.0572	0.1737	0.7422	−0.3976	0.2833	0.9445	0.6719	1.3275						
	**Inverse variance weighted**	**17**	**−0.2794**	**0.1249**	**0.0253**	**−0.5243**	**−0.0346**	**0.7562**	**0.5920**	**0.9660**				17.1075	16.0000	0.3787
	Weighted mode	17	−0.0200	0.2285	0.9314	−0.4678	0.4278	0.9802	0.6264	1.5339						

MR, Mendelian randomization; OR, Odds ratio; PA, pyogenic arthritis; SNP, single nucleotide polymorphism.

### Four bacterial traits are positively correlated with PA

The IVW method found a positive association between four gut bacteria and PA risk: *Adlercreutzia* (OR 1.3210, 95 % CI 1.0181–1.7141, *P*=0.0362, q=0.8612), *Holdemania* (OR 1.2239, 95 % CI 1.0013–1.4960, *P*=0.0485, q=0.5774), *Anaerostipes* (OR 1.3614, 95 % CI 1.0189–1.8191, *P*=0.0369, q=0.7322) and *Butyricimonas* (OR 1.2627, 95 % CI 1.0016–1.5921, *P*=0.0484, q=0.6406), respectively. The results are presented in Table 2 in detail.

**Table 2.** The MR results of four gut microbiota that were identified to be positively correlated with PA

Exposure	Method	nSNP	b	se	pval	lo_ci	up_ci	OR	or_lci95	or_uci95	Egger_intercept	SE	Pval	Q	Q_df	Q_pval
***Adlercreutzia***	MR Egger	12	0.1170	0.6784	0.8665	−1.2127	1.4466	1.1241	0.2974	4.2487	0.0153	0.0630	0.8128			
	Weighted median	12	0.1270	0.1615	0.4314	−0.1894	0.4435	1.1355	0.8274	1.5581						
	**Inverse variance weighted**	**12**	**0.2784**	**0.1329**	**0.0362**	**0.0179**	**0.5389**	**1.3210**	**1.0181**	**1.7141**				14.4972	11.0000	0.2067
	Weighted mode	12	0.1565	0.2482	0.5413	−0.3300	0.6429	1.1694	0.7189	1.9020						
***Anaerostipes***	MR Egger	15	−0.4909	0.4433	0.2881	−1.3598	0.3779	0.6120	0.2567	1.4592	0.0564	0.0295	0.0780			
	Weighted median	15	0.2532	0.2115	0.2311	−0.1612	0.6677	1.2882	0.8511	1.9497						
	**Inverse variance weighted**	**15**	**0.3085**	**0.1479**	**0.0369**	**0.0187**	**0.5983**	**1.3614**	**1.0189**	**1.8191**				10.8895	14.0000	0.6947
	Weighted mode	15	0.2749	0.3513	0.4470	−0.4136	0.9633	1.3164	0.6613	2.6205						
***Butyricimonas***	MR Egger	16	−0.1154	0.4636	0.8070	−1.0240	0.7932	0.8910	0.3592	2.2104	0.0296	0.0380	0.4495			
	Weighted median	16	0.1738	0.1616	0.2820	−0.1429	0.4905	1.1898	0.8669	1.6331						
	**Inverse variance weighted**	**16**	**0.2333**	**0.1182**	**0.0484**	**0.0016**	**0.4650**	**1.2628**	**1.0016**	**1.5921**				14.0511	15.0000	0.5217
	Weighted mode	16	0.0826	0.2699	0.7638	−0.4464	0.6115	1.0861	0.6400	1.8432						
***Holdemania***	MR Egger	18	−0.3637	0.2879	0.2247	−0.9281	0.2007	0.6951	0.3953	1.2222	0.0609	0.0292	0.0535			
	Weighted median	18	0.1885	0.1379	0.1716	−0.0818	0.4589	1.2075	0.9215	1.5823						
	**Inverse variance weighted**	**18**	**0.2021**	**0.1024**	**0.0485**	**0.0013**	**0.4028**	**1.2239**	**1.0013**	**1.4960**				19.3431	17.0000	0.3092
	Weighted mode	18	0.2033	0.2243	0.3775	−0.2364	0.6430	1.2254	0.7895	1.9021						

MR, Mendelian randomization; OR, odds ratio; PA, pyogenic arthritis; SNP, single nucleotide polymorphism.

### Robustness and validity of instrumental variable analysis in causal associations

**Fig. 3.**
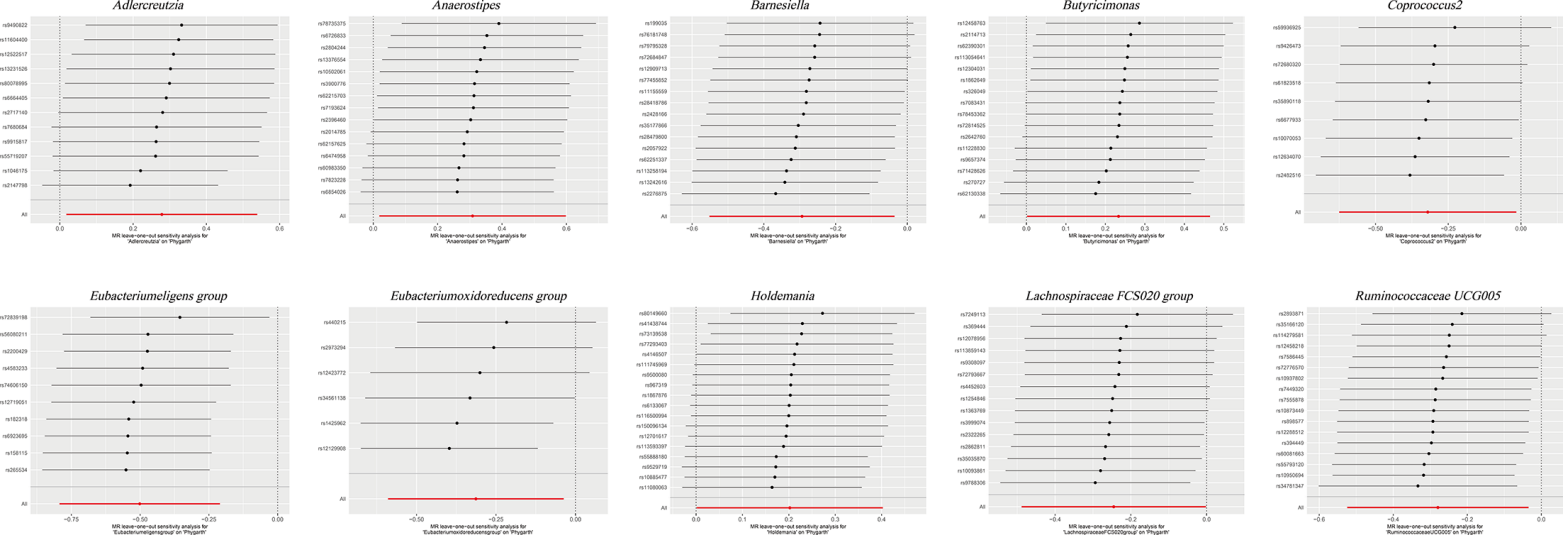
Leave-one-out plots for the causal association between gut microbiota and PA.

**Fig. 4.**
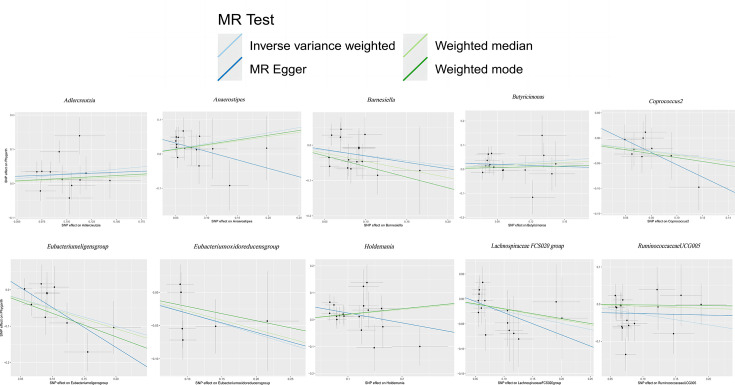
Scatter plots for the causal association between gut microbiota and PA.

The findings from Cochran’s IVW Q test indicated an absence of significant heterogeneity among the IVs (Tables1  2). This conclusion was further substantiated by the leave-one-out sensitivity analysis, which confirmed the robustness of the results (Fig. 3). Visual inspection of the scatter plots (Fig. 4) revealed the presence of potential outliers among the IVs. Nevertheless, the MR-PRESSO analysis did not identify any significant outliers (*P*>0.05; Table S4). Moreover, the MER intercept analysis provided no indication of notable directional horizontal pleiotropy (Tables1  2).

## Discussion

Our results showed that genetically predicted *E. eligens* group, *Barnesiella*, *Coprococcus 2*, *Ruminococcaceae* UCG-005, * E. oxidoreducens* group and *Lachnospiraceae FCS020* group exhibited a negative correlation with the risk of PA, whereas *Adlercreutzia, Holdemania, Anaerostipes* and *Butyricimonas* were positively associated with the risk of PA.

The genetic associations observed in this study reinforce the causal link between gut microbiota and PA, as demonstrated through MR. By leveraging genetic variants as instrumental variables, this analysis minimizes confounding factors and reverse causation, offering robust evidence of microbiota’s influence on PA. Protective genera, such as *E. eligens group* and *Barnesiella*, are likely involved in anti-inflammatory pathways mediated by short-chain fatty acids (SCFAs), immune modulation and gut barrier integrity [23,24]. Conversely, risk-associated genera, such as *Adlercreutzia* and *Holdemania*, may contribute to pro-inflammatory states, potentially exacerbating systemic inflammation and increasing PA susceptibility.

Genus *Coprococcus* has been recognized for its role in producing SCFAs like butyrate, which exhibit anti-inflammatory properties and support gut health. A reduction in *Coprococcus* has been reported to be associated with inflammatory conditions, e.g. inflammatory bowel disease [25]. Similarly, as a common inflammatory arthritis, PA may be inversely associated with genus Coprococcus. In addition, both the *Ruminococcaceae* UCG-005 and *Lachnospiraceae* FCS020 groups belong to the Firmicutes phylum, which are integral to the fermentation of dietary fibres into beneficial SCFAs. The capacity to reduce inflammation and enhance mucosal barrier function may elucidate their inverse correlation with the risk of PA [25]. Genus *Barnesiella* has been demonstrated to be associated with modulating the immune response and inhibiting colonization by pathogenic bacteria, which may account for its negative association with PA [23]. Moreover, the *E. eligens* group and *E. oxidoreducens* group are recognized for their production in SCFAs [24]. SCFAs, including acetate, propionate and butyrate, play a crucial role in regulating the immune system, enhancing the intestinal barrier and reducing systemic inflammation. A decline in these SCFA-producing bacteria could compromise immune homeostasis and barrier function, allowing translocation of bacterial components into the bloodstream [24,26]. Taken together, the above six genera may reduce the risk of PA via both inflammation and immune modulation.

Conversely, our results indicated that *Adlercreutzia*, *Holdemania*, *Anaerostipes* and *Butyricimonas* were positively correlated with the risk of PA, respectively. Genus *Adlercreutzia* has been implicated in metabolizing phytoestrogens, which can influence both immune responses and inflammation [27]. The positive correlation between *Adlercreutzia* and PA might reflect a pro-inflammatory effect under certain conditions. Additionally, *Holdemania* and *Anaerostipes* are known for their roles in metabolizing complex carbohydrates and producing SCFAs [28]. Their association with the increased PA risk suggests that they may have context-dependent effects on inflammation. A prior study demonstrated that an elevated presence of *Holdemania* in the gastrointestinal tract plays a role in triggering inflammatory responses [29]. 
Although *Butyricimonas* is generally associated with butyrate production, it can also contribute to inflammation by disrupting immune regulation or engaging in pathogenic interactions within the gut. Therefore, the current evidence supports the results of our study. These findings emphasize the role of gut microbiota in systemic inflammatory diseases and highlight their potential as targets for innovative microbiota-focused therapies, such as probiotics, dietary interventions or microbial metabolite-based treatments. This study also opens avenues for precision medicine approaches, aiming to modulate gut microbiota composition for the prevention and management of inflammatory conditions like PA. Importantly, our findings underscore the intricate role of the gut microbiome in systematic inflammatory disorders. For example, SCFAs are generally known as general beneficial agents. However, some SCFA-producing bacteria, such as *Holdemania*, can also exacerbate inflammation [29]. It highlights the complex interactions between gut microbes and host immune responses, which emphasize the necessity to further elucidate the specific mechanism behind the casual relationship between gut microbiota and PA. Moreover, as a common local bacterial infectious disease, PA is firstly reported to be associated with an imbalance in gut microbiota composition (human body may function as an interconnected system) in the present study. Therefore, understanding the onset and progression of diseases from a holistic perspective offers novel insights and methodologies for future studies on PA. Similar studies have revealed the significant role of gut microbiota in osteoarthritis (OA). For instance, *Actinobacteria* and *Bifidobacteriaceae* were shown to have protective effects against OA, partially mediated by basal metabolic rate [30]. Additionally, some other taxa, *Gordonibacter* and *Eubacterium* brachy group, were reported to be associated with an increased risk of OA. These results were consistent with our findings, which further demonstrated the pivotal influence from gut microbiome on systemic inflammatory and degenerative diseases [31]. The above insights underscore the potential for microbiota-targeted interventions in managing both OA and PA.

This study identified six genera of gut microbiota—*E. eligens* group, *Barnesiella*, *Coprococcus* 2, *Ruminococcaceae* UCG-005, * E. oxidoreducens* group and *Lachnospiraceae* FCS020 group—that exhibit a negative correlation with the risk of PA, thereby suggesting their protective effects. Conversely, four genera—*Adlercreutzia*, *Holdemania*, *Anaerostipes* and *Butyricimonas*—demonstrated a positive association, indicating their potential role in increasing disease susceptibility. The application of MR analysis provided compelling evidence for a causal relationship between gut microbiota and PA, effectively reducing confounding variables and the possibility of reverse causation. These genetic associations implicate specific bacterial genera in the systemic inflammatory processes that underlie PA, offering critical insights into the disease’s pathogenesis. The protective genera identified in this study could be harnessed for microbiota-targeted therapies, such as probiotics or dietary interventions, to mitigate PA risk. Meanwhile, the genera associated with increased risk could serve as biomarkers for early detection or as therapeutic targets. Future research should prioritize longitudinal studies incorporating direct microbiome profiling to validate these associations, investigate the mechanistic pathways linking gut microbiota to immune responses and inflammation, and extend research to diverse populations to enhance the generalizability of findings and identify population-specific interactions.

Our research boasts several notable strengths. First, it is the inaugural MR study to explore the causal link between gut microbiota and PA. The genetic variation data concerning the gut microbiome is sourced from the most comprehensive GWAS meta-analysis to date, enhancing the reliability of the IVs in the MR analysis. Second, the use of MR analysis to determine the causal relationship between gut microbiota and PA effectively addresses confounding factors and reverse causality challenges typically encountered in causal inference. Third, potential horizontal pleiotropy was rigorously evaluated and excluded through the application of MR-PRESSO and the intercept from MER testing. However, the limitations of our study should also be acknowledged. First, the bacterial taxonomic analysis was confined to the genus level, omitting more granular taxonomic distinctions such as species or strains. Second, to obtain suitable IVs, a less stringent *P*-value threshold of *P*<1.0×10^−5^ was employed, as opposed to the traditional genome-wide significance level of *P*<5×10^−8^ in selecting gut microbiota. Third, the GWAS primarily involved participants of European descent, potentially restricting the generalizability of our findings.

## Conclusions

In summary, this study demonstrates a causal relationship between gut microbiota and PA, with specific protective and risk-associated genera identified. These findings underline the potential of microbiota-targeted interventions, such as probiotics, prebiotics or dietary adjustments, to mitigate PA risk. Clinically, this research emphasizes the need for integrating gut microbiota profiling into inflammatory disease management to identify high-risk individuals and explore personalized treatment strategies.

## Supplementary material

10.1099/jmm.0.002004 Uncited Supplementary Material 1.

## Abbreviations

CI: confidence interval
GWAS: genome-wide association studies
IVs: instrumental variables
IVW: inverse-variance weighted
LD: linkage disequilibrium
MER: MR-Egger regression
MER: MR-egger regression
MR: mendelian randomization
MR-PRESSO: MR pleiotropy residual sum and outlier test
OA: osteoarthritis
OR: odds ratio
PA: pyogenic arthritis
SNPs: single nucleotide polymorphisms
WM: weighted model-based
WME: weighted median estimator
